# Metaphors as models: Towards a typology of metaphor in ancient science

**DOI:** 10.1007/s40656-021-00450-2

**Published:** 2021-08-18

**Authors:** Marcel Humar

**Affiliations:** Department of Classics, Humboldt-Universität zu Berlin, Berlin, Germany

**Keywords:** Ancient science, Metaphors, Typology of metaphors, Biology, Models, Terminology of biology

## Abstract

Metaphors play a crucial role in the understanding of science. Since antiquity, metaphors have been used in technical texts to describe structures unknown or unnamed; besides establishing a terminology of science, metaphors are also important for the expression of concepts. However, a concise terminology to classify metaphors in the language of science has not been established yet. But in the context of studying the history of a science and its concepts, a precise typology of metaphors can be helpful. Metaphors have a lot in common with models in science, as has been observed already. In this paper, therefore, I suggest a typology of metaphor in ancient science to fill this terminological gap by using concepts applied to the classification of models in science, as coined by Rom Harré. I propose to differentiate between homeoconceptual metaphors (with the same conceptual frame between source and target) and paraconceptual metaphors (mapped via a different conceptual frame). Furthermore, functional and structural aspects of metaphors in ancient science are taken into account. Case studies from ancient texts displaying metaphors in ancient science are presented and classified following the outlined typology of metaphors.

## Introduction

Metaphors have been exclusively treated as poetical or rhetorical devices for a long time and sometimes were regarded as a “deviant” form of language (Mooij, 1976, p. 8). In particular, metaphors in science have long been dismissed as unnecessary, improper, and even obscurantist and, hence, deceptive^1^ (for attitudes about metaphors in science, see Haack, 2019, pp. 2050–2052; Hoffman, 1980, pp. 399–400; Montuschi, 2000, p. 278; Locke already declined the use of metaphor in philosophy in his *Essay concerning human understanding*, 1690, Book III, X §34, cf. Clark, 1998). Another point of criticism is rooted in the reductive view that metaphors could be substituted by literal language and are superfluous in a way. This view, however, has changed recently, and it has become clear that metaphors are crucial in science because they are essential constituents of theory^2^ and play an important part in science communication. They also faciliate understanding in the context of science teaching. This quality of metaphors has been emphasized, among others, by Miller (2000, p. 219): “Metaphors are an essential part of scientific creativity because they provide a means for seeking literal descriptions of the world around us. These literal descriptions are scientific theories.” Regarding the concept of metaphors as constituents of theory, also see Boyd (1993). With a view to science communication, the creative use of metaphors especially is sensible when a term for a specific structure or phenomenon does not suffice to describe the object properly or to draw attention to important aspects, or when no other term is available^3^ (pointed out by Martin & Harré, 1982, p. 97 and Sangoi, 2014, p. 78). Hence, metaphors can fill a lexical gap in the vocabulary of scientific language.

In recent years it has also become clear that there are various types of metaphor used in society in everyday language as well as in literary texts. These types range from dead metaphors to metaphors being truly alive. In the context of literary studies and linguistics, we find typologies using terms like ‘dead’, or even metaphors that are named ‘bold’ (in German ‘gewagte Metapher’, see Weinrich, 1966, p. 61) and are characterized by an (alleged) incompatibility between target and source (on these terms, see below); further, we find ‘mixed’ metaphors, which consist of several incongruous metaphorical expressions combined (see Sullivan, 2019). The most detailed typology in linguistics has been suggested by Newmark (1988), who distinguished among ‘dead’, ‘cliché’, ‘stock’, ‘adapted’, ‘recent’, and ‘original’ metaphors (for a critical review of this typology, see Dickins, 2005). We are dealing with dead metaphors when one hardly recognizes the metaphor because of its frequent use (e.g., speaking of a table’s leg or of the mouth of a river); this dichotomy between ‘dead’ and ‘alive’ metaphors has been challenged by Müller (2008).^4^ A cliché metaphor belongs to a group of metaphors that probably have “outlived their usefulness” (Newmark, 1988, p. 107) but can be described as emotive.^5^ Stock metaphors, like dead metaphors, are established and frequently invoked but not overused (one example is “to oil the wheels”; cf. Newmark, 1988, p. 108). If stock metaphors are adapted and personalized in some way, Newmark speaks of ‘adapted metaphors’. Metaphors that are (mostly) anonymously coined and are generally used in the source language can be called ‘recent’ metaphors (metaphorical neologisms). An ‘original’ metaphor is created by a writer or speaker and is neither lexicalized nor adapted.

Comparing all the different disciplines dealing with the theory of metaphor, however, it is surprising that a concise typology of the metaphor in science is still missing. Since metaphors in science belong to the specific terminology of a discipline (e.g., biology, medicine, physics), we cannot use the typology suggested by Newmark, because metaphors, for instance, in science commonly do not become ‘outlived’ or even dead, as every student of science has to learn those terms from the ground up; furthermore, metaphors in science have other functions than metaphors used in poetry or even in everyday language (on the different functions of metaphors in science, see below). As a result, the typologies of metaphors in linguistics or literature have not been extended to metaphors in science. Metaphors in technical literature are described and analyzed in several works, but without a framework that would allow many useful distinctions. Thus, there are many open questions that could be answered by referring to a typology. There are different kinds of metaphors, with different conceptual as well as semantic properties; but are there certain types of metaphor that are primarily used in science? Which types are used in theory building, and which types are convenient for communication? To look into all this, it might be useful to have a typology, especially with regard to questions of how metaphors in science can be coined and, in the history of their use, why they were abandoned or accepted. Boyd (1993, p. 482) notes that “[…] one should expect that when metaphorical language is employed in a scientific context, its function should either lie in the pretheoretical (prescientific?) stages of the development of a discipline, or in the case of more mature sciences, it should lie in the realm of heuristics, pedagogy, or informal exegesis, rather than in the realm of the actual articulation or development of theories.”

To fill the gap of a missing typology mentioned above, I will, as a centerpiece of this article, suggest a typology of metaphors in ancient science, adopting a concept from model theory and adjusting two concepts applied to classify analogical models in science by Harré (1970, 1976). Specifically, the aim is to provide a precise typology of metaphors used in the language of natural philosophy in antiquity.

In this article, the suggested typology is applied to ancient texts, and I will illustrate the different types of metaphor using examples drawn from Aristotle, Theophrastus, Aelianus, and Pliny the Elder. It seems useful to start with ancient texts when presenting the typology because they stand at the beginning of the history of science in Western thought (see, for instance, Mouton, 2013; Zee, 2017). Furthermore, because of their influence, we can easily follow their history in the progression of scientific thought. Difficulties with the proposed typology will also be discussed in the conclusion.

Facing the role of metaphors within the development of scientific concepts (for interesting case studies in cellular biology, see Reynolds, 2018), a typology of metaphors in science can help to understand how and perhaps why concepts (and metaphors used to describe them) have changed over time and why there have been “novel extensions and new elaborations of that ancient metaphor” (Mouton, 2013, p. 313). Thus, a typology helps us first to gain further structured insights into the possibilities of constructing metaphors in ancient science with a focus on different qualities of target and source. Hence, my typology might provide a system to describe the diversity of metaphors used in scientific language. Since metaphors can be considered as cognitive analogies with different degrees of abstraction (on viewing metaphors as analogy, see Sewell, 1964, p. 42 and Gentner et al., 1988, 2001), a typology focusing on ‘conceptual frames’ and the level of abstraction can describe metaphors more properly.

Another way in which my suggested typology might contribute to the understanding of metaphors in ancient science concerns the criticism of metaphors (see my short remarks above): since no detailed typology of the metaphors used in scientific discourse exists, it is unclear whether the criticism mentioned above is applicable to all metaphors in scientific descriptions or only to certain kinds. Another, more general justification of the necessity to classify metaphors in technical texts is that a classification is required for characterization. If we want to understand the different types of metaphor in ancient science, we need to classify them to bring order into the vast amount of metaphors. The need for classification is obvious in biology: we can say few things about animals in general, more so about vertebrates, and more so about mammals, and even more about humans. The same holds true for metaphors: if we classify them according to certain standards, we might get a better understanding of what they are and how they work.

A very loose classification can be found in the study by Boyd (1979), who suggests the existence of two different categories of scientific metaphors with view to their context in which they should be used, namely theory-constructive and pedagogical/exegetical metaphors. The former are used in original scientific thought and newly coined terminology, whereas pedagogical metaphors serve to explain already existing knowledge.

In modern research on metaphors, there has been an attempt to define the function and character of metaphors, which led to a vast amount of literature on this topic. I shall restrict my remarks concerning the function and terminology of metaphors, for my purpose, to necessary aspects only.

## On metaphors

A metaphor links two domains by mapping attributes from one onto the other. Thus, metaphor is an act of transferring. Different terminological conventions have been used to describe these two domains. In this paper, I will use the terms ‘source’ and ‘target’, which are commonly used in cognitive linguistics. The key terms, ‘target’ and ‘source’, were introduced by Lakoff and Johnson (1980). For a general introduction to cognitive metaphor, see Finke et al. (1992) and Kövecses (2002); a short overview can be found in Sangoi (2014, pp. 79‒81).

The source provides the attributes or concepts in a metaphorical statement, while the target accepts them. In short, a metaphor states figuratively that a target is a source. The target is different from, but in a way analogous to, the source. Hence, the metaphor assists people to understand one object or phenomenon (the target) in terms of its resemblance to another (the source).

For instance, the biological metaphor ‘genes are text’ links the source ‘text’ and the target ‘genes’ (on genes and human language, see Shanon, 1978; on metaphors in molecular genetics, see Nelkin, 2001). In keeping with this metaphor, we speak of the *translation* of genes, *transcription* of genes, *proof*-*reading* polymerases, the different *letters of the genetic alphabet* (i.e., the nucleotides), and so on. This metaphorical conceptualization makes it easier to ‘access’ the topic and talk about it as well as invent new theories about the function of genes.

Metaphors are established by focusing on a similarity or analogy between two domains.^6^ However, a metaphor works with a partial overlap of two domains that draw attention to commonalities while ignoring inessential features (Sangoi, 2014, p. 76). Aristotle stated in a famous passage in his *Rhetoric* (Rhet. III 4, 1406b 21‒24) that by describing Achilles metaphorically as a lion, the speaker intends to focus on the Greek hero being brave (also possible: fierce, savage). Achilles, however, does not resemble the lion in walking on four legs or having huge canines or a mane. Hence, the metaphorical expression is not attributing all the qualities of a lion to Achilles. The recipient of that metaphor does not need to have biological (or, at least, anatomical) knowledge about lions, but a cultural knowledge that makes it possible to understand what is emphasized by using this metaphor (on the cultural basis of metaphor, see Quinn, 1991).

Metaphors are not meant to be judged by the same standards as literal statements. They cannot be either true or false because they are not literally claiming an identity between source and target. As Mary McClosky (1964, p. 218) puts it in an often-quoted^7^ passage: “To use truth tests on a metaphorical statement is to take it literally and metaphorical statements are to be taken metaphorically.” On metaphor and truth, see also Mac Cormac (1985, pp. 207‒225).

Instead, metaphors must be evaluated in terms of their aptness (this was already remarked by Aristotle in Rhet. III 2, 1405a 10‒13. On aptness in metaphor, see Tourangeau & Sternberg, 1981). Metaphors are apt if the intended mapping between source and target is easily comprehended. In the example given above, it is apt to describe Achilles as a lion, since the mapping draws attention to the qualities that he has in common with a lion (being fierce and strong). Hence, metaphors provide insight and knowledge about the object they describe.

## Metaphors and models

Metaphors, like models, have a heuristic function. The invention of a metaphor leads us to deeper knowledge about the thing metaphorically described. Or, in cases of newly developed metaphors, they make a hidden likeness come to light. Max Black (1962, p. 37) emphasized that it would be more illuminating “to say the metaphor creates the similarity than to say it formulates some similarity antecedently existing”.^8^ The cognitive dimension of metaphor was already mentioned by Aristotle, who remarked that metaphor “does something which adds knowledge” (Top*.* 140a 9).^9^ The language of science, especially biology,^10^ is particularly rich in metaphors,^11^ as has been noted already (Bronowski, 1978, p. 28; Haraway, 1976; Maasen, 1995; Ouzounis & Mazière, 2006; Perelman, 1969). As Gell (1983, p. 83) notes: “The use of metaphor and analogical thinking is crucial to theory building in biology, in fact possibly theory building in biology is no more than the development of useful metaphors –which is much more crucial than measurement. The shared use of metaphors in art and in science seems to me possibly the cement that keeps the two together.”^12^ However, a concise terminology to describe the structure or character of different metaphors in science has still not been elaborated. As mentioned in the introduction, I intend to use an adapted terminology of model theory to develop a typology of metaphors.

Why use the terminology of models? Models in science are, like metaphors, a fundamental part of the process of scientific discovery,^13^ understanding, and, of course, teaching (Harrison & Treagust, 2000). Through models, scientists represent ideas about objects or processes within the natural world.^14^ The term ‘model’ (and the verb ‘modeling’) is differently used, and, especially with view to science, it remains unclear what exactly the word ‘model’ means (as noted by Leatherdale, 1974, pp. 40‒43 and Beckner, 1968, pp. 33‒36). However, there is an extensive literature on the use and limits of models in science (an overview is found in Leatherdale, 1974) and science education (Cameron, 2002; Mayer, 1979; Sutton, 1978). But then, what is a ‘model’? A general definition is given by Marvin Minsky (1965, p. 45): “We use the term “model” in the following sense: To an observer B, an object A* is a model of an object A to the extent that B can use A* to answer questions that interest him about A. The model relation is inherently ternary. Any attempt to suppress the role of the intentions of the investigator B leads to circular definitions or to ambiguities about “essential features” and the like.”^15^ And, what makes a model? In his book *Allgemeine Modelltheorie* (general model theory), Herbert Stachowiak (1973, pp. 131‒133) described the fundamental properties of a model as follows: there is an aspect of mapping (models are always models of something), an aspect of reduction^16^ (models do not represent all of the attributes of the thing they are a model of), and an aspect of pragmatism (models are created for a certain purpose,^17^ such as teaching).

Thus, especially with view to the last two points, models generally can be described as limited representations^18^ of reality with a certain purpose (Hesse (1966, p. 9) called models an “imperfect copy”). A scientific model aims at the representation, visual or theoretical, of systems, such as empirical objects, particular phenomena, and processes, physical as well as chemical or biological. A model always displays an analogy by which an easier understanding is achieved. A scientific model could be a diagram or chart, a physical model like a cell made of plastic (both mainly perceived visually), or even a computer program or mathematical law^19^ (both rather theoretical). In addition to making objects and processes easier to understand, models can be used to predict what might happen in the future. So, for instance, a model of a heart with tubes filled with liquid illustrating the blood flow can be used to predict what will happen if one or two of the tubes transporting the blood are clogged. Hence, models provide heuristic functions. In particular, models provide simplified ways to understand ideas and relationships, develop new hypotheses, and make predictions.^20^ However, models are always limited (or reduced) in the sense that a model cannot represent a system completely. Otherwise, it would be the system itself.^21^

The limitation can concern the attention to detail (models cannot include all details of the objects that they represent) or the accuracy (models need to be simple enough to communicate concepts effectively and often sacrifice accuracy to achieve this). Further, models are approximative constructions: these approximations provided by the model are not exact, so predictions based on them may be different from what is observed. Max Black’s (1962) monograph on *Models and metaphors* established a relationship between metaphors and models that has since been developed by others in the field. What both metaphor and model at first sight share is the concept of analogy^22^ and similarity (Giere (2010, p. 269) described similarity as “the basic relationship between models and the world”).

This fact is also stressed by Soskice & Harré (1995, p. 302), who regard models as a non-linguistic analogue of metaphor: “A metaphor, we have said, is a figure of speech; a model is a non-linguistic analogue. An object or state of affairs is said to be a model when it is viewed in terms of its relationship to some other object or state of affairs. The relationship of model and metaphor is this: if we use the image of a fluid to explicate the supposed action of the electrical energy, we say that the fluid is functioning as a model for our conception of the nature of electricity. If, however, we then go on to speak of the ‘rate of flow’ of an ‘electrical current’, we are using metaphorical language based on the fluid model.”

Further, models help us acquire knowledge, whenever they make things unknown or less understood, accessible in terms (or models) we can understand. Metaphors, like models, are also limited in the sense that they ignore many aspects of the target in the process of highlighting other features.^23^ What I suggest in this paper is a typology of metaphor using concepts usually applied to models.^24^ The aim is to provide a more precise typology of metaphors in science, as mentioned in the introduction.

## Towards a typology of metaphor

### The ‘structural’ and ‘functional’ aspect of models and metaphors

As already noted, models are limited representations of real objects or processes. Hence, they do not show perfect resemblance. Instead, some properties of the phenomenon being modeled are emphasized, while others are deemphasized or ignored. Models often focus on either the structure or function of the subject to be modeled. Therefore, especially within the German literature on models in science, a terminology that differentiates (though not exclusively^25^) between models of function and models of structure has been established (Lay, 1973, p. 522 and especially in biology, for instance, Halbach, 1974). For instance, a model of the human heart that is intended to visualize the position of blood vessels, the arteries and veins, the heart chambers, and the proportions of the different parts has to be a model of structure (a ‘structural’ model). A structural model primarily illustrates proportions, forms, and haptic aspects. Such a model is less suited to clarifying the function of the heart (blood pressure, systolic and diastolic phases, etc.). The reason is that the model is constructed of solid, and hence ‘immovable’, compartments or compartments that are displayed dissected.

On the other hand, if a model’s purpose is to explain the function of the heart, it might be made of soft tubes and filled with liquid (e.g., red and blue ink). But in creating such a model, it might not be necessary or practical to preserve the same proportions of the parts with the materials available or to have the plastic tubes closely resemble arteries and veins in appearance. Of course, in some cases mixed models are possible that demonstrate the function of an object while also sharing its structure to some degree.

The distinction between structural and functional models has an analogue in metaphors in biological terminology, since metaphors can likewise be used to draw attention to either structural or functional similarities, in this case between source and target. Thus, I use the terms ‘structural’ and ‘functional’ metaphors to describe more precisely the typology of metaphor in science. For instance, when the Greek botanist Theophrastus, a student of Aristotle, described tree sap as a ‘tear’ (*dákruon,* δάκρυον; see HP 2.7.11.6; 2.7.7. 9; 4.4.12; 4.7.2.5; 7.6.3.13), he was employing a structural metaphor because the source and the target share the same form rather than function. The function of a human tear is to lubricate the eye (and show emotions); the function of tree sap or resin is to seal ‘wounds’ caused by invading insects. Theophrastus, in describing the tree sap as a tear, focused on the structure, which can be described as drop-shaped (I will provide more examples below).

Functional metaphors that draw attention to a similarity in function between a source and a target are also found in ancient scientific literature. For example, in describing the trunk of an elephant in his *History of animals* (*Historia animalium*, HA), Aristotle probably invented^26^ a metaphor borrowed from architecture: “One part of the nose is the cartilaginous *diáphragma*, [while] the *ochéteuma* is empty” (HA I 11, 492b 16; all translations from ancient texts are mine unless otherwise noted).

The first part of the nose, the *diáphragma* (διάφραγμα), refers to the nasal septum, which is soft, or, in Aristotle’s view, cartilaginous. The word ‘diaphragm’ was used before Aristotle to describe a wall within a building (Thucydides Hist. 1.133.1). The function of a wall in the source domain of ‘architecture’ to stabilize and separate carries over to the target domain, except that the nasal septum is soft and limber, while the wall of a building is solid and rigid. The second word, the *ochéteuma* (ὀχέτευμα), or nasal cavity, also comes from architecture. In Herodotus, we find the verb *ocheteúō* with the meaning of “leading water through a pipe” (Hist. 2.99.20). Therefore, Aristotle was plausibly conceiving it not merely as a cavity but as a conduit. Hence, both terms are functional metaphors. The trunk is paralleled with an architectural building or construction.

That Aristotle focused on function when inventing these terms becomes clear from the passage that immediately follows: “The nose of the elephant is long and strong, and the animal *uses it like* a hand [ὥσπερ χειρί]; for by means of this organ it *draws objects towards it*, and takes hold of them, and introduces its food into its mouth, whether liquid or dry food, and it is the only living creature that does so” (HA I 11, 492b 17 f., translation after Thompson slightly modified, my italics). As can be seen from the text, the trunk functions as a pipeline; and this aspect of functions is emphasized by Aristotle. Hence, this passage seems to be an ‘explanation’ for choosing the terms *diáphragma* and *ochéteuma*.

However, the distinction between ‘functional’ and ‘structural’ metaphors is not clear-cut. For example, Homer described the outer body of crustaceans as a *thórax* (θώραξ), which usually referred to the body protection of men in battle (Hom. *Iliad* 4.133; 5.100). In HA IV 2, 601a 13, Aristotle followed the same naming convention: “In fact, the frontal part is more pointed, and the thorax is much broader in the lobster than in the spiny lobster, and the body in general is smoother and fuller of flesh.” Aristotle was using the same metaphor several centuries later to describe the mid-section of lobsters and spiny lobsters, and modern biology still uses this term to describe crustacean anatomy today. I treat this as both a functional and structural metaphor because the functional aspect (protective) and the structural aspect (hard, sclerotized) are both in focus.^27^

In botany, Theophrastus uses an interesting ‘animal’ metaphor to describe the structure of leaves that also has both functional and structural aspects. Recognizing that for some properties of plants no names are available, he consciously borrows some terms from the animal kingdom: “Tendons and blood vessels lack names of their own. They borrow [their names] from the parts of animals because of their resemblance” (HP 1.2.3). In this passage, he uses the terms ‘tendons’ (*ines*, ἶνες) and ‘blood vessels’ (*phlébes*, φλέβες) to refer to the leaf veins, which they closely resemble (τῇ ὁμοιότητι). The veins of animals and leaves both have the same structure (narrowing ramifications) and the same function (leading a fluid through the organ). Hence, the analogy conveyed by the metaphor potentially has both structural and functional aspects. However, it is not so obvious that Theophrastus and his contemporaries know that leaf veins have this function. But the structural resemblance between both ‘organs’ is obvious.

These few examples demonstrate how a typology of metaphor differentiating between structural and functional aspects can help to describe metaphors more precisely regarding their resemblance between target and source. However, other useful distinctions drawn from the terminology of models can also be applied to metaphors relating to the degree of abstraction or the types of systems represented.

In the following section, I will give a brief overview of two important terms introduced by Rom Harré (1970) and demonstrate how they can be applied with slight modifications to the typology of metaphor.

### A typology of models

The taxonomy of models used by Harré (1970) is based upon the ways a model can relate to the object being modeled concerning the degree of similarity and abstraction. I will focus on two main categories of models: *homeomorphs* and *paramorphs*.^28^

Models whose source and target share nearly all features because the models depict the same object in the natural world are called homeomorphs (Harré, 1970, p. 40; Harré, 1976, p. 24; Harré, 2004, pp. 8 − 9) or homeomorphic model. For instance, a heart made of plastic is an example of a homeomorphic model, because the plastic heart models a real heart, while a real heart is also the source of the model (likewise, all models of human organs are homeomorphs. A model of a kidney models a kidney, the model of a lung models a lung, and so on). A homeomorph focuses on a certain aspect of the system represented and does this with high resemblance. In other words: there is a very large overlap between the features of the model and the object or process being modeled. Regarding the cognitive potential and epistemic value of such a model, one can say that it is easy to understand what object is modeled and what its structure or qualities are. For instance, a homeomorphic model of an insect is clearly recognizable and provides information about the modeled insect immediately. Hence, the intuitive comprehension of that model is high.

A paramorph (or paramorphic model) relates to an object in a different manner than the source of the model (Harré, 1976, p. 24; Harré, 2004, pp. 8 − 9); paramorphs are highly abstract and do not resemble the shape of the object or process modeled. With a paramorphic model, the source that is modeled from is quite different from the subject being modeled. For example, using a computer (*source*) to model the cognitive activity of the human brain (*target*) is a paramorph. Thus, a paramorphic model involves the application of ideas, examples, or thoughts from another domain or source to the subject modeled. To put it simply, the overlap between model and the object/process modeled by it is small, the resemblance is not quite obvious, and the quantity of common features is low. Additionally, the types of features they have in common differ (concrete vs. abstract features). The intuitive comprehension of such a model is lower than in homeomorphs, since one cannot easily access the information provided by the model. For instance, a mathematical model cannot be understood without detailed knowledge concerning the process being modeled.

Paramorphs are needed if the processes or objects one wishes to model raise difficulties because they are relatively unknown or the mechanisms to be modeled are too complex or dangerous to work with. An example of paramorphic models in modern science might be the key–lock principle in enzymes. First introduced by Emil Fischer, this theoretical model illustrates the bonding between enzymes and specific substrates. Enzymes and other chemical substances are completely different from keys or locks. Thus, this kind of model can be called a paramorph. For instance, a person not trained in molecular biology would likely have trouble understanding what the model of competitive inhibition in enzymes means. As can be seen from this example, metaphors are the epistemic *precursor* of paramorphic models, because the inventor of that model first must think about his modeled object metaphorically (the enzyme *is* a key).^29^ Or, as Brown (2003, p. 26) put it: “Models are extended metaphors that have the potential to guide thinking about a system under investigation, suggesting new directions for research.”^30^ But this only holds true for paramorphic models because homeomorphs are only imperfect copies of an object (differing, for instance, in size, color, material) but with a high resemblance and, hence, hardly can suggest new directions for research.

These two terms of scientific model theory—homeomorphic and paramorphic —can be used to describe metaphors more precisely, although there are differences between models and metaphors. For instance, metaphors *always* express something different from the source, and therefore metaphors cannot be homeomorphs in the stronger sense of being models. Nevertheless, I want to suggest that the terminology describing models is also apt for describing metaphors, which is demonstrated in the following section.

### A model typology of metaphors

Since metaphors are conceptual and cognitive tools, I will modify the terms presented above a little bit. A metaphor can be called *homeoconceptual* if the source and the target share a relatively close resemblance concerning their structure or function and are related to the same *conceptual frame*. The recipient of that kind of metaphor can easily infer information about the object or process described by the metaphorical expression because there is a high degree of overlap between the features of source and target within the same conceptual frame. The similarities are immediately cognitively accessible; hence, it is easy to visualize the target in terms of the source.

For instance, the ‘genes are texts’ metaphor could be described as a homeoconceptual, since the linked sequence of molecules that comprise a gene shows a high resemblance to letters placed in a specific order (text); and both ‘texts’ contain certain information. Likewise, the metaphor ‘molecular scissors’ for restriction enzymes is a homeoconceptual functional metaphor, since these enzymes ‘cut’ (which means destroy or cut into pieces) the DNA into fragments and, hence, share the same conceptual frame (but, of course, they do not look like scissors) because within the scientific discourse molecules are often presented like ‘tools’ (e.g., protein kinases like switches that turn other proteins on or off, or channel proteins in the nerve cells). This metaphor provides insight into cutting enzymes to people unfamiliar with these molecules.

Cognitive accessibility is much lower for *paraconceptual* metaphors, which show a higher level of abstraction because target and source are not mapped via the same conceptual frame (or the underlying metaphorical concept has not been established yet). The author of this kind of metaphor combines, maybe for the first time, different frames that have not been connected so far or do not share a common concept within the terminology of science. The meaning of the metaphorical term ‘chaperone molecule’, for instance, is quite opaque without possessing detailed knowledge of molecular biology.^31^ This metaphor is highly abstract or notional and, therefore, not easily accessible (and a concept that treats molecules metaphorically as human beings has not been established in scientific discourse). Further, the conceptual frame is different (proteins do not ‘behave’ in a way and, hence, they do not need a chaperone).

By categorizing metaphors along a functional/structural dimension within the homeoconceptual dimension and having the paraconceptual dimension on the other side, we have a typology consisting of a total of three distinct types (see the table below). I will illustrate each of these types with examples from ancient technical texts in the following sections. The functional/structural division is left undifferentiated in the paraconceptual dimension because of the high degree of abstractness, which compounds a distinct attribution to one category.

**Table:** 

	Homeoconceptual	Paraconceptual
Functional	Source and target are mapped via sharing the same ‘function’ and being related to the same conceptual frame	Source and target are mapped via a different and complex conceptual frame. They show a high level of abstraction
Structural	Source and target are mapped via sharing the same ‘structure’ and being related to the same conceptual frame	

## Case studies

### Homeoconceptual structural metaphor

The aforementioned example of tree sap as a ‘tear’ is a homeoconceptual structural metaphor: A tear (*dákruon*) and a drop of sap or resin have the same form, which can be easily identified as the property the metaphor is drawing attention to. They also share the same conceptual frame (every small amount of fluid running from a surface forms a drop-like structure). The leaf veins (*ines*, *phlébes*) mentioned in the same passage can also be described as homeoconceptual since they belong to the same conceptual frame (vascular structures in a body).

We find several other examples of this type of metaphor in ancient science. In Aristotle’s account of crustaceans, he uses the word *chēlé* (χηλή) to refer to the crab’s or lobster’s claw (e.g., in HA IV 3, 527b 5; PA IV 8, 684a 27). The word is used, most likely in its original sense, also for the cloven hoofs of cattle (see, for instance, Euripides Bacchae 619, Ion 1242). Since the cloven hoof’s primary function is associated with walking, while for claws it is clasping, this is an example of a homeoconceptual structural metaphor (they do not share the same function but have a similar structure and belong to the same conceptual frame: extremities of the body). Hence, because of the similar structure of target and source, the intention of the metaphor is transparent, and very little cognitive effort is required to make sense of it. A further example can be found in the anatomy of crustaceans: Aristotle described the lobster in a very detailed manner. Within this passage, we find two homeoconceptual metaphors:

And in general, the facial part is more pointed, and the ‘thorax’ is much broader than in the languste; and the whole body is more fleshy and softer. Of the eight feet, four are forked at the tip and four are not. The region of the ‘neck’ [περὶ τὸν τράχηλον] as it is called is divided externally into five portions, and the sixth is the broad region at the end, which has five flaps (HA IV 2, 526b 4‒9, translation after A. L. Peck).

The *thorax* (or breastplate, see above) is a homeoconceptual metaphor because both target and source belong to the same conceptual frame (protective parts on the body). Likewise, their structure is very similar as well as their function.

While further describing the body of the lobster, Aristotle appeared to be following the common usage of his time by describing the abdomen as a ‘neck’ (*tráchēlos*).^32^ The word generally referred to a neck (Herodotus Hist. 2.40 and Plut. Art. 29; for *tráchēlos* describing especially the neck of animals, see, for instance, Xen. Equation 1.8) or any narrowing structure, but in this case, Aristotle was using it as a metaphor for another structure of the lobster’s body: the abdomen. Since the abdomen does not share any functions with the neck but is related to the same concept (name of body parts, found near the head) and has the same structure, we might speak of a homeoconceptual structural metaphor.

Another metaphor of that type is the term ‘horns’ (*kérata* or *keraia*); non-metaphorically, this term refers to the appendages on the head of ruminants. By describing the ‘horns’ (*keraia*) of the *strombōdē*, a group of snails (mentioned by Aristotle in HA IV 4, 528b 24), Aristotle used a homeoconceptual structural metaphor because these structures on their fore-head (today named *antennae*) have the same position and appearance, and the term is mapped via the same concept (any appendage on the head of an animal can be named ‘horn’). The same holds true for the ‘horns’ of the crustaceans (*kérata*, see HA IV, 2 526a 31) and the ‘horns’ (*keraia*) of insects (both a kind of antennae or ‘feelers’) named in HA IV, 7 532a 26.

In his work *De partibus animalium* (PA), Aristotle refers to the inner shell of the calamari as a *xiphos* (ξίφος), literally a sword.^33^ This term is a homeoconceptual structural metaphor since the calamari’s inner shell does not have any of the functions of a sword (cutting or defense) but closely resembles a sword in form and structure. The conceptual frame is the same since any sharpened and pointy structure can (mentally) represent a sword or knife.

Another homeoconceptual structural metaphor found in ancient texts is the ‘head’ (*kephalé*) of plants, which refers to a kind of bulb on the root or lower part of the stem. In his *Historia plantarum* (HP 1.6.9.4‒6), for instance, Theophrastus described such structures as being the origin of certain roots sprouting from them. We can regard this term as a homeoconceptual structural metaphor since every rounded form on a body (of plants and animals) can be regarded as a head (in the case of the ‘head’ of the plants with the roots as its ‘hairs’). Another example: Theophrastus uses the term *ioulos* (ἴουλος, e.g., in HP 3.5.5; 3.7.3) to describe the specific structure of certain flowers with a type of clustered growth known as a ‘catkins’. Several plants bear catkins, including willows, birches, and oaks. The word *ioulos* literally refers to the first growth of the whiskers^34^ and beard (as in Hom. Od. 11.319). Since plants do not possess hair in a strict sense but the structure Theophrastus called *ioulos* closely resembles a beard in form and shares the same concept (outgrowth that covers the surface of a body), this is a homeoconceptual structural metaphor.

A person lacking an education in biology and has never seen the inner shell of a calamari, or the claw of a crab, or feelers of a snail, or the bulbs or catkins of a plant is nevertheless able to get a sense of the structure of these organs mediated via the metaphors mentioned above. Since they share the same conceptual frame (homeoconceptual aspect) regarding their form (structural aspect), it is easy to grasp an idea of their structure and which similarity the metaphor draws attention to.

### Homeoconceptual functional metaphor

In a short passage in *On breathing* (*De respiratione* X, 476a 2‒5), Aristoteles explains the term *ptéruges* (πτέρυγες) describing the fins of fish: “The group of the so-called Selachia and all other footless animals have gills. All fish are footless, and [the limbs] they have their name [*pterúgion*] from their similarity to wings [*ptérux*].” The word *ptéron*, rather describing the wings of birds, here in its diminutive form refers to the fin of fish.^35^ Since wings and fins share the same function—to navigate within a certain habitat (air, water)—and show less similarity concerning their structure (wings are feathered, fins are built by fin rays with taut skin between those rays), this is more a functional metaphor. Because of the same conceptual frame (body parts for moving), this metaphor is homeoconceptual.

In describing the specific behavior of apes and guenons regarding their storage of food within their mouth, the Roman natural encyclopedist Pliny the Elder (Hist. nat. 10.93) uses an interesting and obvious metaphor to describe the cheek pouch of those mammals: “The group of guenons and apes store the food in the vault [*thesaurus*] of the jaws, and after a while they take it out with their hands for chewing it.” Since a vault and the space in the mouth of those animals share the same function (storing something for later use), this is a homeoconceptual functional metaphor focusing more on the functional aspect. Interestingly, in common language, the metaphor describing this structure has changed to a more structural metaphor. In English, we speak of a *pouch*; in German, we use the word *Backentasche* to designate a space in the mouth used for storing food. This example shows how in the history of terminology we can use the structural/functional typology of metaphors to describe the change in concepts when analyzing the terms. Another example of a homeoconceptual functional metaphor can be found in the *ochéteuma* presented above describing the elephant’s nostrils on one side, and on the other side, referring to an artificial waterway in architecture (the mutual concept that links both structures is a spatial one: any hollow structure in a body or wall could be named a ‘duct’ or ‘channel’). Probably a more anatomically correct metaphor could be the term ‘pipette’, since the nose of the elephant is used to soak up some water and dip it into the elephant’s mouth.

All these metaphors are understandable because they are *self-explanatory* if the object metaphorical depicted is in front of one’s own eyes because of sharing the same conceptual frame. Hence, the person to whom these metaphors are presented has immediate cognitive access to them.

### Paraconceptual metaphors

Facing the examples given above, one might suggest that in structural or functional metaphors, there must always be a homeoconceptual aspect, because if two objects have a similar structure or function, they must look similar. Nevertheless, we find examples in the terminology of ancient science, which can be described as *paraconceptual* because there is a large difference in appearance between the target and the source of the metaphor so that whatever subtle resemblance does exist between them is not immediately obvious. This is especially true when the resemblance is made by combining *different conceptual frames*, and if the linking concept is unknown, access to the intention of the metaphor is blocked and it will fail to be understood without further explanation.

In Aristotle’s *Generation of animals* (GA 744b 10), we find the metaphor of nature as a good householder (οἰκονόμος ἀγαθός). To describe nature as a household^36^ might be a paraconceptual metaphor because a person interpreting this metaphor needs a certain amount of knowledge about natural ‘housekeeping’ processes to grasp the intended analogy (the complex processes in nature and the processes in a household do not belong to the same conceptual frame, which makes the metaphor not self-explanatory). An extensive analysis of the concept ‘nature as a household’ in antiquity is found in Leunissen (2010).

Aristotle sometimes also described nature as a craftsman using the verb ‘δημιουργέωʼ (*dēmiourgéō*, to work at, fabricate; see PA I 5, 645a 9: ἡ δημιουργήσασα φύσις). So, for instance, in his work *On the progression of animals* (*De incessu animalium* 711a 17–19), he wrote: “The reason for this is because nature makes nothing in vain [ἡ φύσις οὐδὲν δημιουργεῖ μάτην], as has been said before, but everything [is made] to improve the things it belongs to.” Concepts original to housekeeping like storage and waste *can* be transferred to ecological processes,^37^ and nature, as a creating force, brings forward organisms like a craftsman builds a house. According to Kullmann (1998, p. 240), this metaphor is Aristotelian in essence. Since nature and craftsmen do not share any relevant similarity other than ‘producing’ something and are mapped via different *concepts* (and a conceptual frame that links them has not been established yet), this is a paraconceptual metaphor.

Within Aristotle’s thermodynamical physiology, we find another paraconceptual metaphor: the term *pépsis* (πέψις, concoction or cooking) meaning a process of digestion caused by the vital heat of the body (see, e.g., PA 651a 20–23; see also Mete. 379b 18–19 and GA 725a 11). This process serves two effects: it softens the food and prepares it for nourishment by separating it into its components (digestion of food) but is also applied to the production of semen. The term has its own history, as Lloyd (1966, pp. 204–205) stated: “Originally used of the ripening of fruit, then of cooking and digestion it came to be applied to a wide range of physiological processes.” According to this statement, the word *pépsis* is used as a metaphor by Aristotle, who did not have any insight into biochemical processes and, therefore, had to find a way to express his thoughts about the processes in question. Because both target and source are mapped via different concepts (cooking/household on the one side, processes within the body on the other), this is a paraconceptual metaphor.

Another paraconceptual metaphor is the term ψυχή (*psychē*), which originally referred to the soul, but which has also been used to describe the adult butterfly. In Aristotle’s *History of Animals*, we find the first known explanation of the term:

The so-called *psychē* [καλούμεναι ψυχαί] is generated from caterpillars which grow on green leaves, chiefly leaves of the raphanus, which some call crambe or cabbage. At first it is less than a grain of millet, it then grows into a small grub, and in three days it is a tiny caterpillar. After this it grows on and on, and becomes quiescent and changes its shape, and is now called a chrysalis. The outer shell is hard, and the chrysalis moves if you touch it. It attaches itself by cobweb-like filaments, and is unfurnished with mouth or any other apparent organ. After a little while the outer covering bursts asunder, and out flies the winged creature that we call the *psychē* (HA V 19, 551a 13–24; translation by Thompson 1910, slightly modified).^38^

Again, we find a rather abstract concept: butterflies are humans. The source (the human soul) and the target (the adult butterfly) share a similarity that is not obvious at first sight. The mapping is constructed via two very different conceptual frames that have to be primarily understood. There are similarities in structure: First, a dead body and the larval state of butterflies (*chrysallis*) are both hard (*sklerós*). Second, the soul leaves the body by (metaphorically) flying away (*ekpétomai*), much as the adult butterfly leaves the now dead shell of the chrysalis (the verb *ekpétomai* is used by Homer Il. 16.856 and 22.362 to describe the soul leaving the dead body). Since both target and source are mapped with a very different conceptual frame that presupposes several aspects, this is paraconceptual metaphor.

Another example of a paraconceptual metaphor can be found in the description of the skin shed by a snake (or insects^39^) after the process of molting. Snakes, when the old skin is outgrown, regularly shed their skin by rubbing against rough surfaces. In antiquity, the product of this process was called ‘the old age’, *gêras* (γῆρας), as exemplified by Aelianus: “When a snake sloughs its old skin, it does so at the beginning of spring, then is the time when it purges away the mists over its eyes and the dullness of its sight and what I may call the ‘old age’ (γῆρας) of its eyes” (Aelian, *De natura animalium* 9.16).

Since this metaphorical expression does not rely on a direct similarity between age and the skin that was shed, and both target and source belong to a different conceptual frame (age belongs to time while skin is a structure), this expression can be classified as a paraconceptual metaphor. There is no functional or structural connection between target and source. An explanation^40^ for this metaphor might be that the metaphor is constructed by the similar quality of the eyes during both processes, aging in men and molting in snakes. The snake’s eyes before and during the process become dull,^41^ much as the eyes of old men. Interestingly, the Neoplatonic philosopher Porphyrios (third century CE) uses in *Contra Christianos* frg. 88, line 16, another metaphor to describe the slough of a serpent after molting: *thórax* (θώραξ), meaning breastplate.

Another example of a paraconceptual metaphor can be found in the description of the fruit of the mallow (*Malva silvestris*). The seed type of this particular plant is called the ʻcakeʼ (*plakoûs*, πλακοῦς) in a fragment of Phainias of Eresos (Frg. 44 Wehrli); also in Theophrastus a similar adjective (πλακουντώδης) is found to describe the fruit of a sweet grass in HP 4.10.4. Both metaphors are paraconceptual because, to my knowledge, nowhere in ancient technical texts are structures of plants described as a product of pastry (hence, we have two different conceptual frames, and there is no metaphorical concept established that connects target and source). Further, a conceptual frame for metaphorical terms in plants already exists: plants are animals (see the next case).

My last example is drawn, again, from Theophrastean botany. In his *Historia plantarum* (HP 7.3.2.7‒14), Theophrastus described a certain type of plant called *gymnospérmata* (γυμνοσπέρματα),^42^ which literally means ‘with naked seeds’. The term probably refers to a certain type of fruit that is not contained within a capsule or pod. This term is an example of a paraconceptual metaphor. Since the seeds, clearly identified by Theophrastus, are not enclosed in a capsule or pod, they appear to be ‘naked’. Naked literally means ‘having no clothes’, which is generally applied only to humans (or in the largest sense to objects we expect to be clothed^43^). Due to the high level of cognitive dissonance (plants do not wear clothes; ‘naked’ only results from the comparison with other structures), this is a paraconceptual metaphor. The metaphor might become more comprehensible if the *gymnospérmata* are visually compared with plants bearing seeds in a seed pouch (in Greek called *ellobospérmata*, ἐλλοβοσπέρματα), but taken by itself, the metaphor is not easily comprehensible because of the conceptual gap between source and target. The structures of plants are often described metaphorically with terms borrowed from animal anatomy (this was already the case in the tendons and blood vessels; see above). While the first examples were easily accessible due to their homeoconceptual character, in this example the underlying concept ‘plants are animals’ (on this metaphorical concept in botanical terminology, see Humar, 2019) must first be understood in order to realize that the term *gymnospérmata* refers to plants with seeds without flesh lying around. Theophrastus seems to have been aware of the high cognitive dissonance of his metaphor: After the passage that names the different types of seeds, he adds a list of examples; this list with plants belonging to the different types is necessary to enable understanding. In this way, he secures understanding by means of examples.

What all these paraconceptual metaphors have in common is that they do not immediately provide a way to understand how they work and what they are referring to because of 1) a large difference between target and source and 2) the existence of different underlying conceptual frames that must be understood before the metaphor becomes accessible.

## Conclusion

Since research on metaphors in science, their development, as well as their modification requires a concise typology, we are in need of a conceptual and typological framework to describe and classify metaphors. I have argued that the typology used to describe models as ‘paramorphic’ and ‘homeomorphic’ is also applicable to metaphors in ancient science and serves as a systematic tool for developing a classification of metaphors in technical texts from antiquity. Especially keeping in mind the degree of conceptual resemblance between target and source*,* this typology of metaphors, first differentiating between structural and functional aspects, and further differentiating between homeoconceptual or paraconceptual sub-types, can lead to a deeper understanding of how metaphors in ancient science are built and provide a framework for further research.

In the following passage, I want to discuss briefly why this typology could be helpful and why we should not focus simply on the aptness of metaphors in science. I will start with the second point: Since metaphors are more or less apt, one might suggest that ‘aptness’ could play a role in classifying metaphors. However, the aptness of a metaphor differs greatly depending on the background of the person interpreting the metaphorical term. In particular, it makes a difference if experts or laymen are exposed to a term. For instance, describing the inner shell of a calamari metaphorically as a ‘sword’ might be apt for both groups of persons, but to speak of the ‘naked seeds’ (*gymnospérmata*) of plants, the ‘old age’ (*gêras*) of snakes, or the ‘cooking’ (*pépsis*) in the body challenges the imagination of laymen, while experts in a certain field may have easier access to those paraconceptual metaphors. Therefore, ‘aptness’ is not a reliable and suitable criterion to classify metaphors in scientific language because aptness is a property of the relation between a metaphor and an individual mind. Following a study conducted in 1985 by Katz et al., a metaphor (in general, not only in science) is thought to be comprehensible if the receiver understands its intended meaning. Katz et al., (1985, pp. 377–378) stated: “Metaphors rated as relatively good are easy to interpret, have only a few interpretations that are particularly salient, are easy to image, and have subjects and topics that are highly related. In short, it appears that better metaphors are those that can be labeled as being more readily interpretable.”

With the distinction between homeoconceptual and paraconceptual metaphors, we have a terminology at hand that allows to specify that statement and explain why a certain metaphor is “relatively good” and how “highly related” could be more specified. This raises further thoughts regarding the criticism of metaphors in science: As mentioned in the introduction, metaphors have been criticized by several authors (from antiquity to modern times). But it is difficult to evaluate whether criticisms of the use of metaphors in science are valid without distinguishing between different types of metaphors and their functions. So, maybe, the criticism of metaphors may have been based mainly on *paraconceptual* metaphors, while *homeoconceptual* metaphors were not discussed in a negative way.^44^

By examining metaphors in ancient technical texts, one can observe that some persisted over centuries, while others disappeared. This might be linked to their level of abstraction as well as to their (same or different) conceptual frames between source and target. At this point, the typology might be helpful to explain the history of metaphors using a precise terminology: homeoconceptual metaphors were readily comprehensible because of their shared conceptual frame and were therefore more readily transmitted from generation to generation. Even today we speak of the *thórax* in crustaceans and the *veins* (*phlébes*) of leaves and *tears* of trees (probably also because the terms reference structural as well as functional aspects of their source). We still name the inner shell of the calamari the *gladius* (sword); in French, the name for a lobster’s abdomen is still *col* (‘neck’), and in ‘flying’ fish the conceptual frame connecting wings and fins is still present.

Paraconceptual metaphors show a high level of abstraction, which may have inhibited their rate of adoption.^45^ For instance, the term we use today to describe the skin that has been shed by a snake is the ‘slough’,^46^ and hence a new structural homeoconceptual (conceptual frame: putting something off) has been coined; so, *gêras* was not successful. We now use the word *gymnospérmata* in a completely different sense (see note 42 above), and we changed the *psychē* into the (non-metaphorical) butterfly. The disappearance of the term ʻcakeʼ in the history of botany could be explained by the fact that it cannot be assigned to the more dominant metaphorical concept (plants are animals) and because of its high abstraction: it is too paraconceptual; therefore, the term has not been established. And without the theoretical framework developed by Haeckel, the metaphor of *nature as household* might also have disappeared in the history of biology. Furthermore, no terms are present in today’s biological terminology that refer to biochemical processes as ‘cooking’.

Hence, the suggested typology of metaphor might be useful if addressing the question why some metaphors are still in use today, and why some metaphors were not preserved in the history of biological terminology. This would make possible what Donald Schön has already demanded, namely to understand the development of a natural science and its theories or insights from the development of its metaphors. Schön (1963, p. 192) remarked^47^: “There is here the possibility for a new kind of inquiry–an intellectual history which would consider not the manifest content of theories, but the development of their underlying metaphors.”

Besides historical aspects, *didactic* or textual aspects can also be illuminated by the typology: Only the paraconceptual metaphors require further explanations to secure the understanding (e.g., a list of examples is given after the term *gymnospérmata* is introduced, a detailed explanation concerning the lifecycle of the *psychē*). In the case of homeoconceptual metaphors, the respective author seems to assume that the terms are understandable due to their common underlying, evident concept. This observation might give a hint that the ancient authors who used metaphors sometimes were aware of the obscure character of their metaphors.

Since I have chosen only metaphors from ancient technical texts to examine the possible application of my suggested typology, it would be necessary to look at metaphors used in science in the various historical periods and traditions after antiquity. I lightly touched on this with my examples from modern biology in the introduction but will leave that for further research.

## Footnotes

1 Anderson (1964, p. 54) wrote that especially in the modern age, with new advances in science, the metaphor was “classified as an embellishment designed to dupe the unwary”.

2 Metaphors provide access to concepts that would otherwise not be accessible (Hesse, 1966; Sangoi, 2014, p. 78), and they furthermore have heuristic and theory constitutive value.

3 This use of metaphor was already stressed by Aristotle in his *Rhetoric* (Rhet. III 2, 1405a 34‒37).

4 Dead metaphors in texts can even be activated by promotive contexts. On this, see Landheer (2002).

5 Newmark (1988, p. 107) presents the following example: “The County school will in effect become not a backwater, but a breakthrough in educational development which will set trends for the future.”

6 According to Black (1962) and his *interactive view* of metaphors, it would be more precise to say that a metaphor *creates* a similarity rather than that “it formulates some similarity antecedently existing” (Black, 1955, p. 285). For an attempt to further develop Black’s ideas, see Indurkhya (1998). For my considerations, I take this focus on similarities to be the main motivation for the use of metaphors in science (nevertheless, this view on metaphor has been disputed by several authors; see, for instance, the overview in Lewis, 1996). For “theory-constitutive scientific metaphors”, see Boyd (1979, p. 362).

7 For example, in Radman (1997, p. 48) and Leatherdale (1974, p. 133).

8 A similar remark is made by Soskice & Harré (1995, p. 290). See also Camac & Glucksberg (1984).

9 See also Rhet. III 10, 1410b 15–19. Aristotle’s view of metaphor was close to the modern view outlined above describing metaphors as a more cognitive than linguistic tool. And Theophrastus, the famous student and companion of Aristotle, mentioned in his *Enquiry into plants* (*Historia plantarum*, HP) the usefulness of analogy within the language of natural philosophy; on the usefulness of analogy, he said: “And again, if in some cases analogy ought to be considered (for instance, an analogy presented in animals), we must keep this also in view; and in that case, we must of course make the closest resemblances and the most perfectly developed examples our standard” (HP 1.1.5; Translation after A. Hort). However, Aristotle was also critical of using metaphors in natural philosophy (especially in Top. 139b 32–35. On metaphor in Aristotle, see Coughlin, 2013; Driscoll, 2012; Gastaldi, 1993. For an overview of Aristotle’s critique of metaphor, see Coughlin, 2013 and Marcos, 2012, pp. 78‒79).

10 Brown (2003, p. 159) has argued that “biology today reveals more forcefully than any other area of science the essential role of metaphor in scientific reasoning and communication”.

11 In biology, for instance, the metaphorical terms ecological *niche*, *mirror* neurons, and family *tree* are only a few examples to mention. On the frequent use of metaphors within the scientific language, see Weinrich (1976, p. 311) and Roelcke (^3^^2010^, p. 75).

12 Note the metaphors he used in the statement.

13 Harré (2004, p. 78): “[…] the proper use of models is the very basis of scientific thinking.” It is obvious that the growth of biological understanding was particularly promoted by the creation of models (e.g., the famous Watson/Crick model of DNA, the model of the cell as an organism, and the model of nature as household introduced by E. Haeckel). The most influential metaphor in science today might be the brain-as-computer metaphor particularly influencing cognitive psychology. On this, see Fernandez-Duque & Johnson (1999). For a treatment of models that stresses their importance in theory construction, see Harré (1976). For the development of models within the philosophy of science, see Bailer-Jones (1999, 2009) and Portides (2008).

14 The word ‘model’ and the concept seem to have been introduced within the realm of physics; probably the first mention of a model in the modern sense comes from James C. Maxwell (1831‒1879). He described the ‘physical analogy’ as follows: “By physical analogy I mean that partial resemblance between the laws of a science and the laws of another science which makes one of the two sciences serve to illustrate the other” (Maxwell, 1890, Vol. I, p. 156). On the introduction of models in science, see Leatherdale (1974).

15 See also Giere (2010, p. 269): “Agents (1) intend; (2) to use model, M; (3) to represent a part of the world, W; (4) for some purpose, P.”

16 The term ‘reduction’ does not mean that models necessarily have to be simpler than the thing being modeled. The reduction is actually in the relation between the model and the thing being modeled rather than in the model per se. Indeed, teachers use real-world examples to model abstract concepts all the time, and these real-world examples are typically more complex in terms of physical properties, numbers of parts, and things like that than the abstract concepts they are being used to explain. These models are still only approximations that leave out certain details, even if they are more complex than the objects they are modeling.

17 In Stachowiak’s words, “Abbildungsmerkmal”, “Verkürzungsmerkmal”, and “Pragmatisches Merkmal”.

18 This holds true for physical models as well as mental models (on this, see Norman, 1983, p. 8). Further, models in science can be understood as dynamic. That means some models are changed with new findings or theories in science; thus, scientists respond to new discoveries or results by changing their models.

19 An example in modern biology might be the Lotka–Volterra competition modeling or the Rosenzweig–MacArthur model in a predator–prey relationship within an ecosystem.

20 The heuristic value of models is partially reappraised by Beckner (1968, p. 33).

21 As Lattmann (2016, p. 216) recently put it: “Nonetheless, as important as models are for *describing* reality, it is not the case that they *are* ‘reality’. Rather they *represent* reality and thus, have a categorically different mode of being. And they not only represent reality, but they represent reality merely partially […]” (italics in original).

22 See Sewell (1964, p. 42): “In metaphor the mind sees and expresses an analogy.” The same view is expressed in Gentner (1983a, p. 162): “Many (perhaps most) metaphors are predominantly relational comparisons, and are thus, essentially analogies.” Metaphor and analogy are coupled together also in Perelman (1969). Aristotle in Top*.* 140a 11‒12 stresses that metaphor is used on the account of similarity (*homoiótēs*). On models and analogies, Hutten (1967, p. 60) remarked: “The model works by means of analogy.” On the connection between models and analogies, see also Hesse (1966) and Canguilhem (1963). On the importance of analogy in science, see the remark of the physicist Oppenheimer (1956, p. 130): “Science is an immensely creative enriching experience; and it is full of novelty and exploration; and it is in order to get to these that analogy is an indispensable instrument.”

23 For instance, the metaphors *argument is war* and *argument is a journey* described by Lakoff and Johnson (1980) highlight different aspects. Using the metaphor *argument is war* enhances the adversarial nature of argument but ignores the fact that argument often needs an ordered and organized development of a topic under discussion. However, the metaphor *argument is a journey* emphasizes the progressive character of argument and hides any confrontational aspects. Max Black conceived of the ‘limitation’ aspect of metaphors as a *filter*. Metaphors work as a filter by emphasizing aspects while ignoring others; see Black (1955, p. 289). For a critique of Black’s term ‘filtering’, see Soskice & Harré (1995, p. 292).

24 For the view of scientific models in terms of metaphors in the history of science, see Bailer-Jones (2000).

25 Other types of models are the model of thought (‘Gedankenmodell’), models of behavior (‘Verhaltensmodell’), and mathematical models. These types are not further discussed here because for my suggested terminology of metaphors, both types named above suffice.

26 The terms discussed below are first found in Aristotle. Of course, this does not exclude the possibility that writers before Aristotle also used these terms. Nevertheless, facing the fact that Aristotle was one of the first who invented a terminology for biology, we can assume that terms that are not attested to someone else before him are his invention. The Peripatetic Demetrius who lived in the 3^rd^ Century CE remarked that Aristotle invented a lot of terms; see Dem. *Peri hermeneias* 97 and 144.

27 As a definite structural metaphor, the word *thórax* is used to describe the slough of snakes after molting; see Porph. Chr. 88. Even in modern biology, we find a metaphorical term concerning the process of molting of the cuticle in reptiles or insects: the process is called *ecdyosis*, which is drawn from the Geek verb *ekdúō* meaning ‘to undress’. The hormone, which controls all these processes, is called *ecdysone*. For another metaphor describing the slough of snakes, see below.

28 Another category is the *isomorph model*, which is characterized by a complete correlation between model and modeled object; since in metaphors *source* and *target* never show complete congruence, this type is not applicable for a classification of metaphors and therefore not considered in this paper.

29 This is another important function of metaphor in science.

30 On models as extended metaphors, see also Kittay (1987, p. 7). On a model being itself metaphorical, see Brown (2003, pp. 21 − 22). See also Gentner (1983b).

31 Molecular chaperones are proteins that assist the folding or unfolding and the assembly or disassembly of other macromolecular structures such as proteins. Therefore, they ‘help’ other molecules to perform their normal biological functions. On chaperone molecules, see Brown (2003, p. 152).

32 A non-metaphorical term referring to the neck or any narrowed structure or passage is ἰσθμός; see Herodotus Hist. 6.36 and 7.22.

33 In modern biology, the inner shell of squids is also called a *gladius* (lat. sword).

34 The metaphorical link is still active in today’s popular terminology: catkin, Kätzchen (German).

35 This metaphorical use of the word πτερύγιον can also be found in *De incessu animalium* 709b 9‒11: “Animals also which fly and those which swim, fly by straightening and bending their wings [πτέρυγας] and swim with their fins [πτερυγίοις], some fish having four fins and others […], having two fins […]” (translation after E. S. Forster).

36 The whole discipline of ecology is founded on this metaphor. The word ‘ecology’ derives from the Greek word for house, household, or habitat (oikos, οἶκος), and the root of the suffix applied to sciences in general (*lógos*, λόγος) and as coined by Ernst Haeckel (1834–1919) in 1866 in his *Generelle Morphologie der Organismen*.

37 Another paraconceptual metaphor in modern ecology might be the term ecological *niche* because this metaphor is not a spatial metaphor (as one could assume) but a more complex metaphor.

38 On the *psychē*, also see Theophrastus HP 2.4.4 and Plutarch Moralia 636 c. The lifecycles of holometabolic insects in general are described in Pliny Hist. nat. 11.41.

39 See Aristotle HA VII, 17 601a 1–2.

40 I owe this remark to Alexander Fürst von Lieven.

41 This is because during shedding snakes produce a special fluid to help separate the old skin from the new. This fluid sometimes causes an opaque or blue appearance to the eye cap, a specially adapted scale on the eye.

42 The modern term *gymnosperms* today is used differently from the ancient term and refers to the group of conifers, cycads, *Ginkgo biloba*, and Gnetales.

43 For example, in the case of the naked mole-rat (*Heterocephalus glaber*), which is hairless, the metaphor is more transparent because of the obvious analogy between fur and clothing, and the fact that most rodents are furry and, hence, we expect the naked mole-rat to be ‘clothed’ by fur.

44 It is beyond the scope of this paper to look at the criticism of metaphors in the history of science and examine if mainly paraconceptual metaphors have been declined.

45 This aspect is also relevant when metaphors serve as a medium for public communication of complex scientific subjects. On this aspect of metaphor, see, for instance, Ouzounis & Mazière (2006).

46 In German, the term *Natternhemd* (the snake’s *chemise*) is used.

47 Also quoted in Mouton (2013).
